# Supplementary material for: A gene regulatory network for neural induction
Source: eLife. 2023 Mar 3;12:e73189. doi: 10.7554/eLife.73189 (PMC10038663; doi:10.7554/eLife.73189)
Supplement: Supplementary file 2. [file elife-73189-supp2.pdf]

A gene regulatory network for neural induction: I. Dynamics of transcriptional responses.

Supplementary Material

Katherine E. Trevers,[1,7,9] Hui-Chun Lu,[1,7] Youwen Yang,[1,3,8] Alexandre Thiery,[2,8] Anna C. Strobl,[1] Claire Anderson,[1] Božena Pálinkášová,[1] Nidia M. M. de Oliveira,[1] Irene M. de Almeida,[1] Mohsin A. F. Khan,[1] Natalia Moncaut,[1,4] Nicholas M. Luscombe,[5,6] Leslie Dale,[1] Andrea Streit,[2] Claudio D. Stern[1,10,*]

[1]Department of Cell and Developmental Biology, University College London, London WC1E 6BT, United Kingdom.
[2]Centre for Craniofacial and Regenerative Biology, King's College London, London SE1 9RT, United Kingdom.
[3]Present address: School of Cardiovascular Medicine & Sciences, King's College London, London, SE5 9NU, United Kingdom.
[4]Present address: Cancer Research UK Manchester Institute, The University of Manchester, Alderley Park, SK10 4TG, United Kingdom.
[5] The Francis Crick Institute, London, UK.
[6] UCL Genetics Institute, Department of Genetics, Environment and Evolution, University College London, London, UK
[7]These authors contributed equally.
[8]These authors contributed equally.
[9]Present address: Research Department of Pathology, UCL Cancer Institute, Paul O'Gorman Building, 72 Huntley Street, London, WC1E 6DD, UK
[10]Lead contact
*Correspondence: c.stern@ucl.ac.uk

0h

29 components
206 interactions

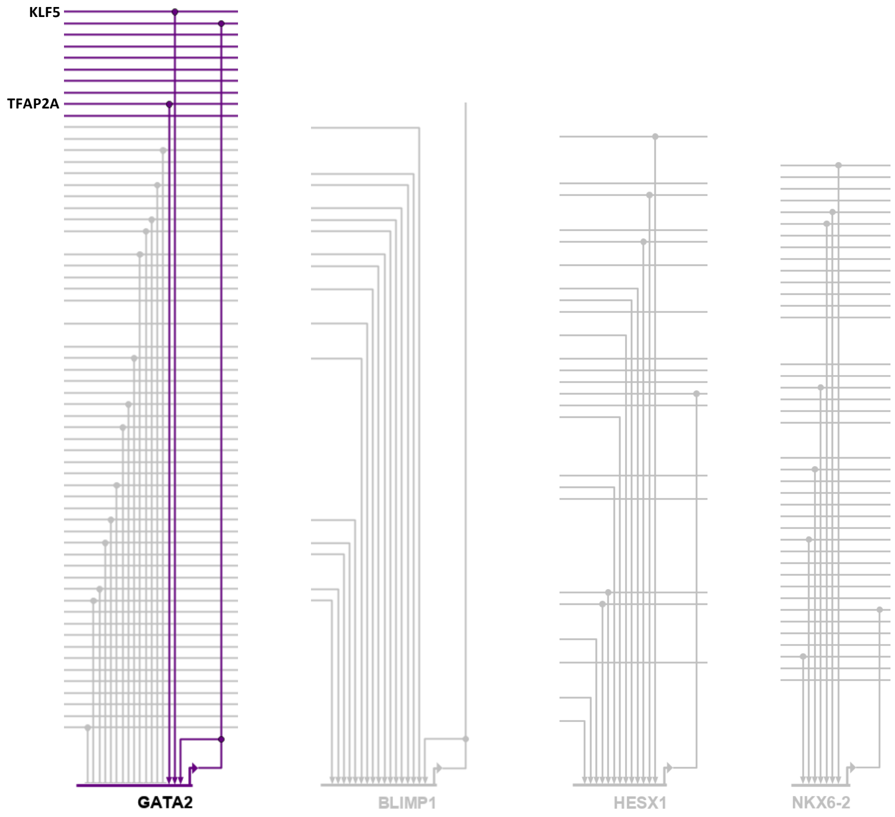

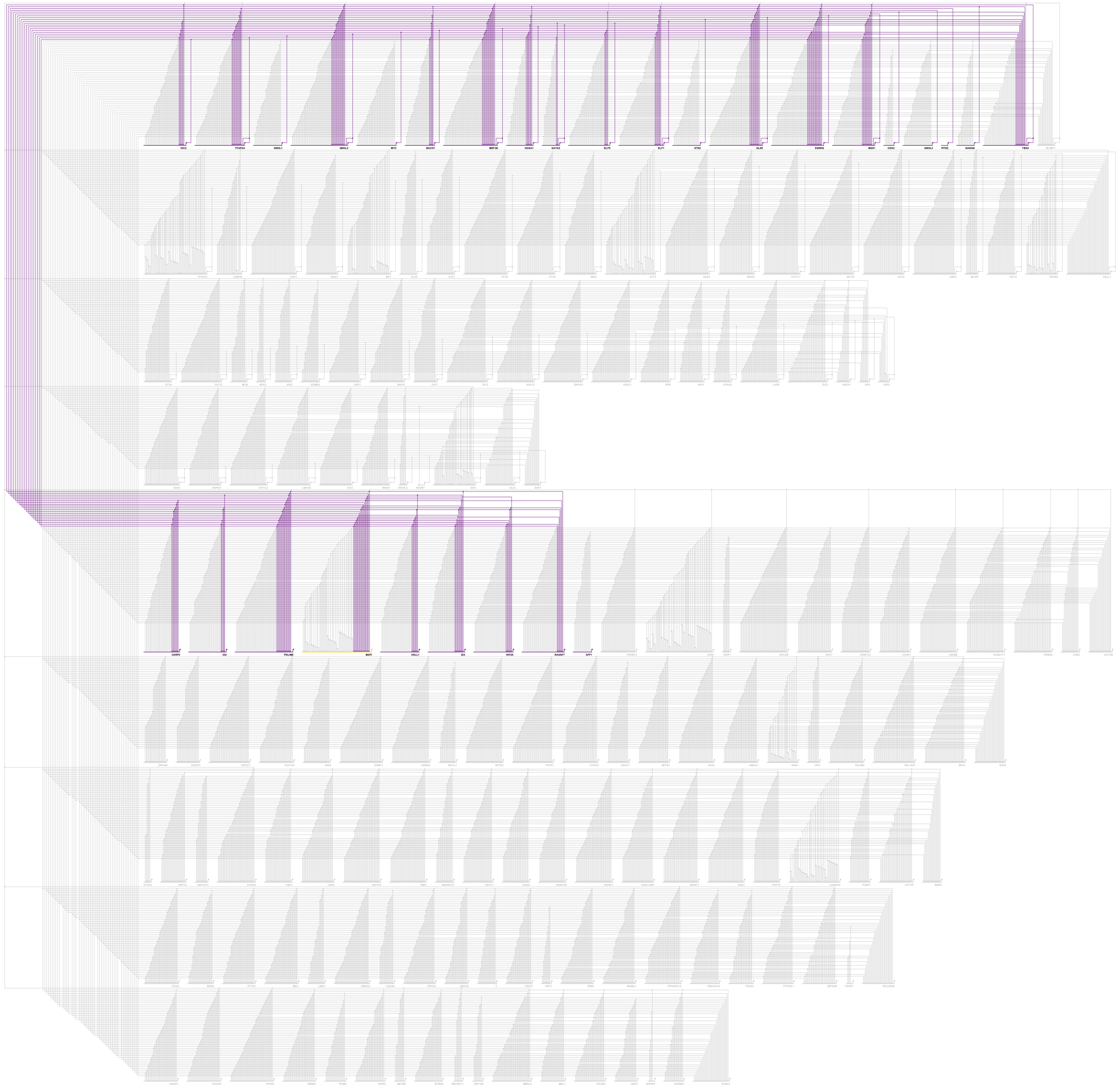

0, 1, 3, 5, 7, 9, 12h

1h

28 components
172 interactions

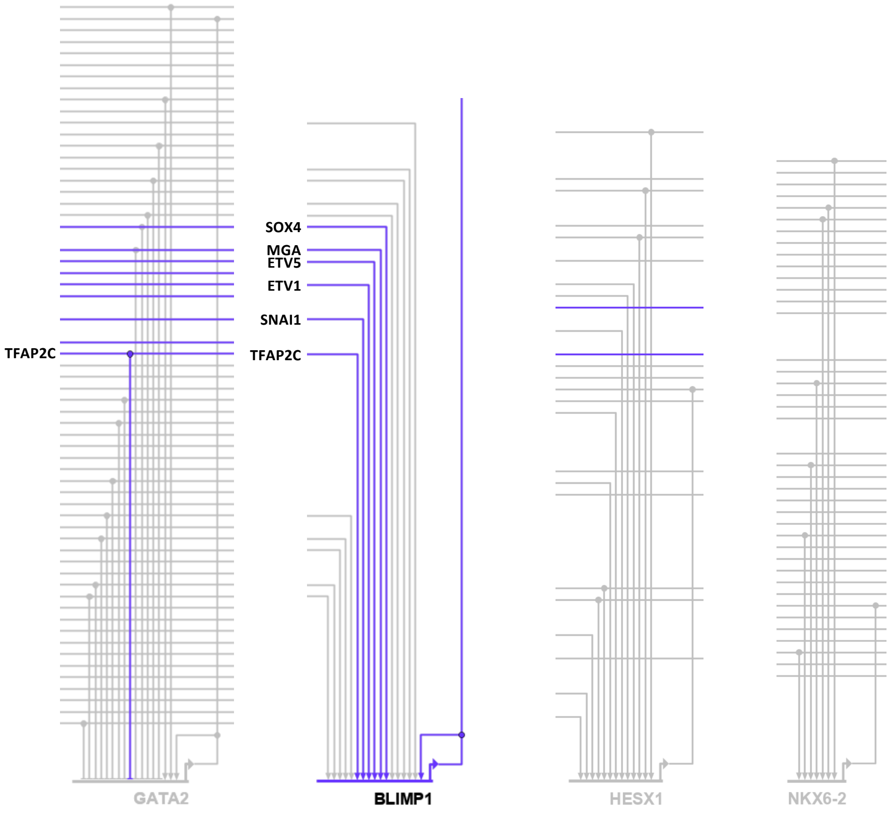

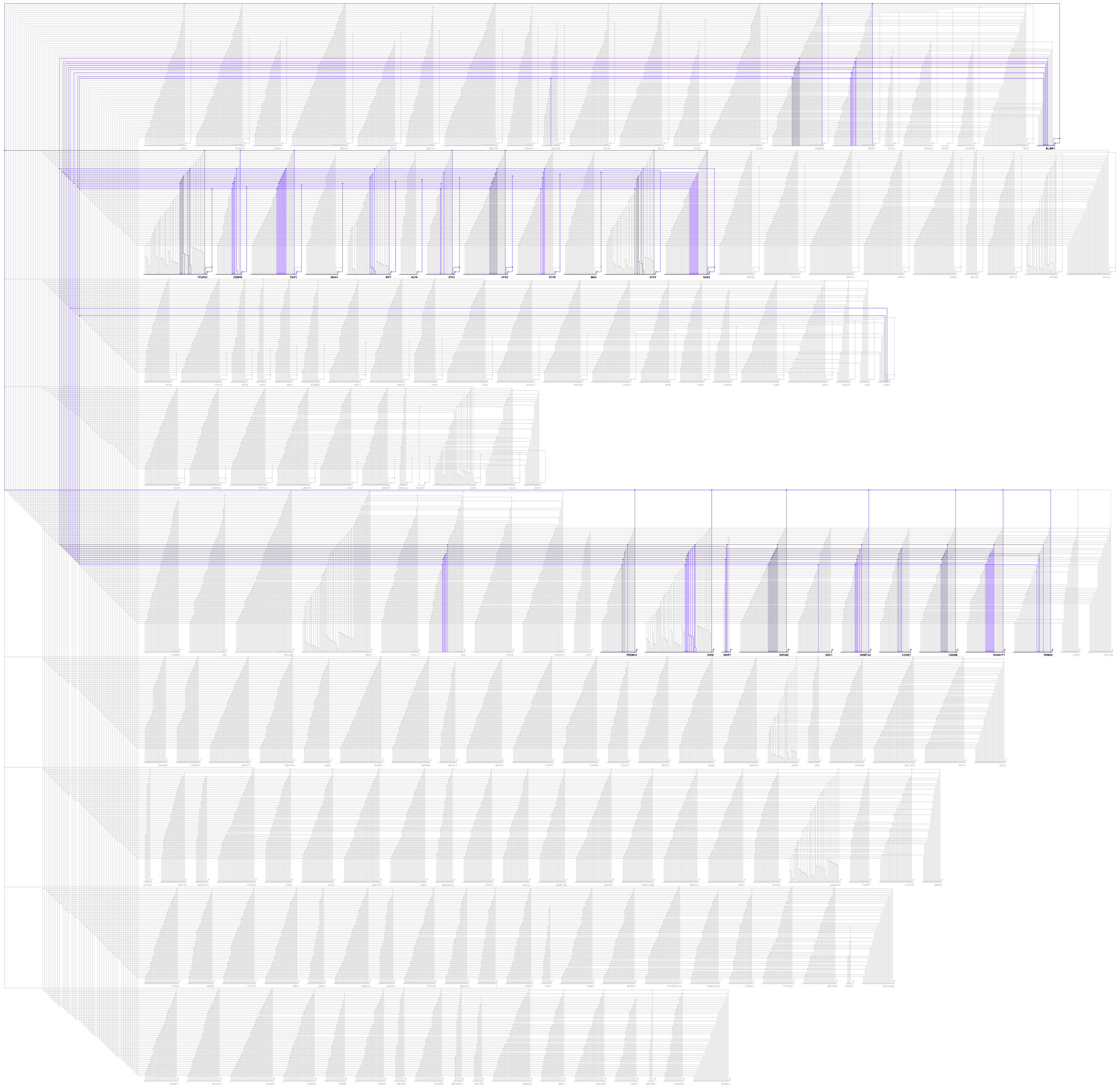

0, 1, 3, 5, 7, 9, 12h

3h

109 components
1,682 interactions

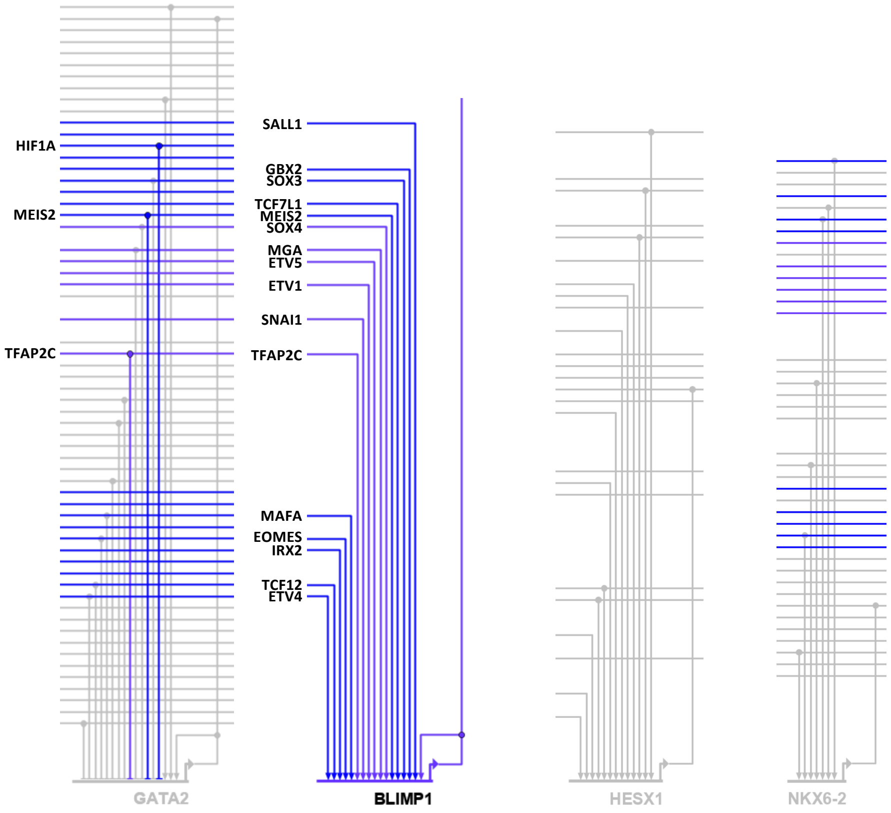

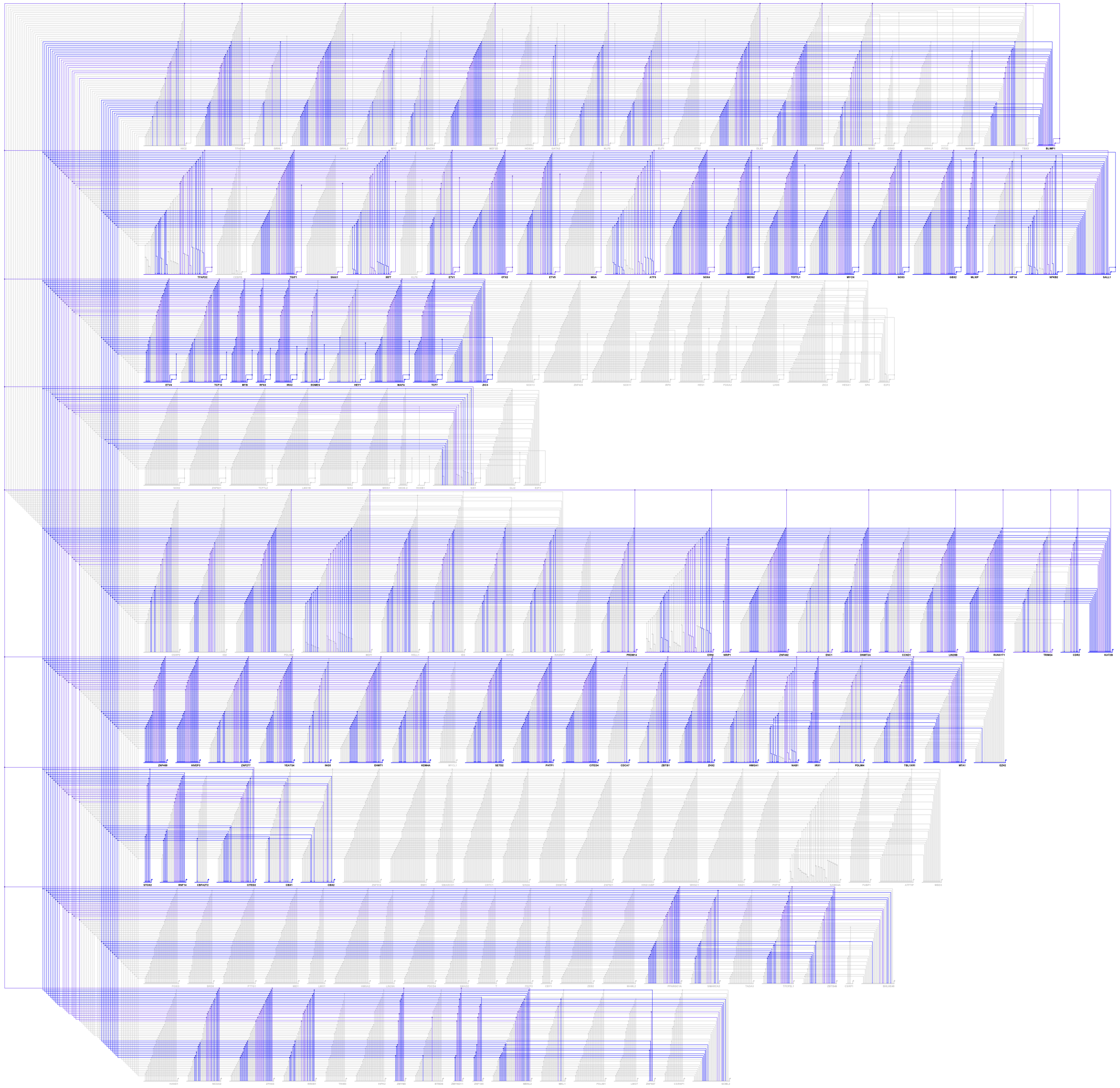

0, 1, 3, 5, 7, 9, 12h

5h

105 components
1,607 interactions

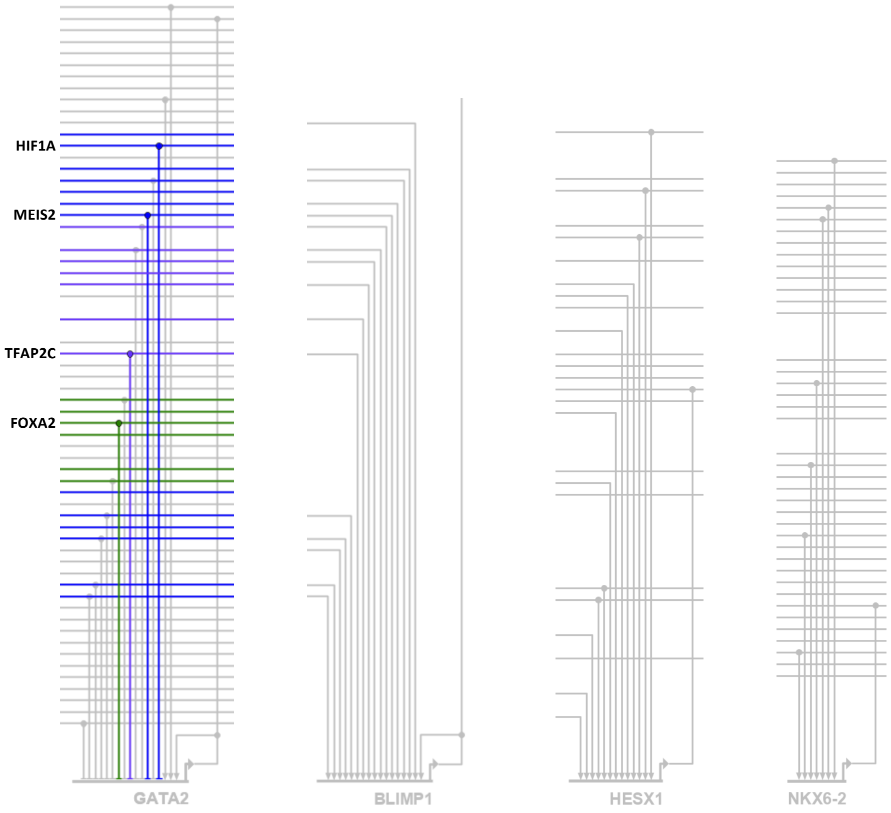

0, 1, 3, 5, 7, 9, 12h

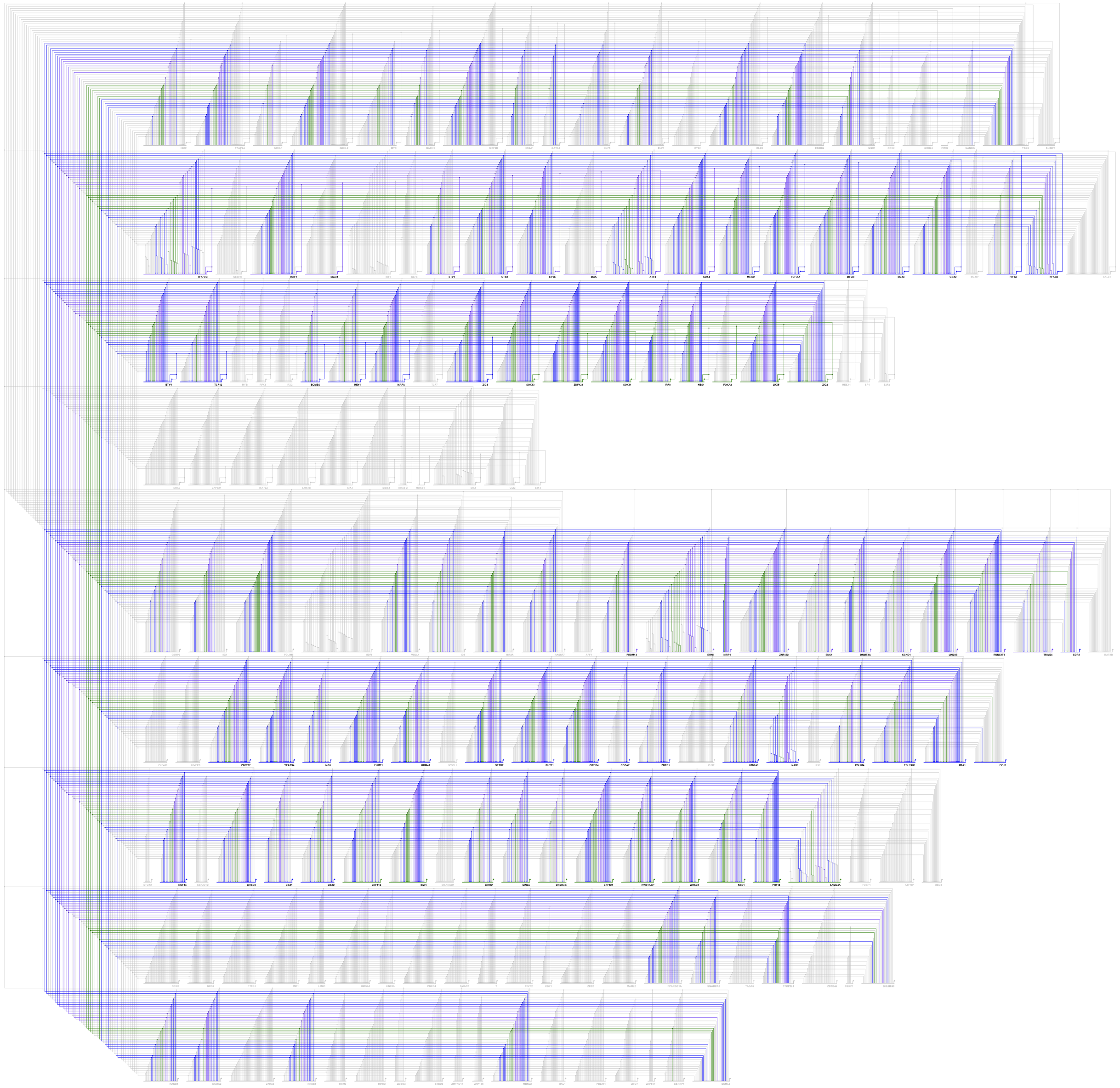

7h

122 components
2,628 interactions

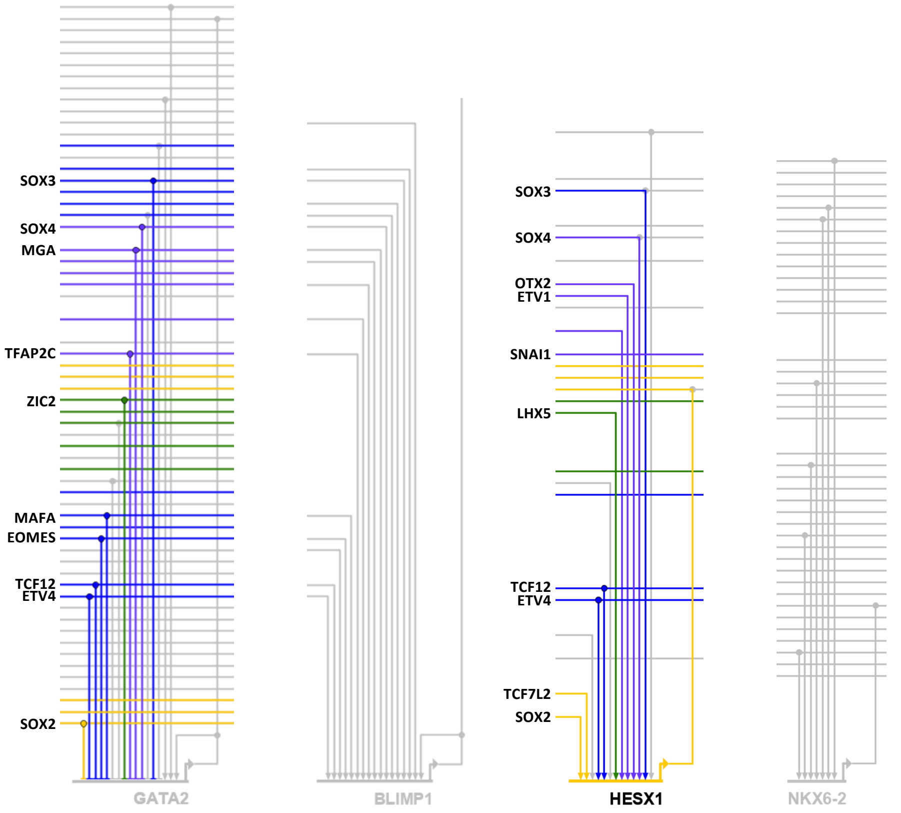

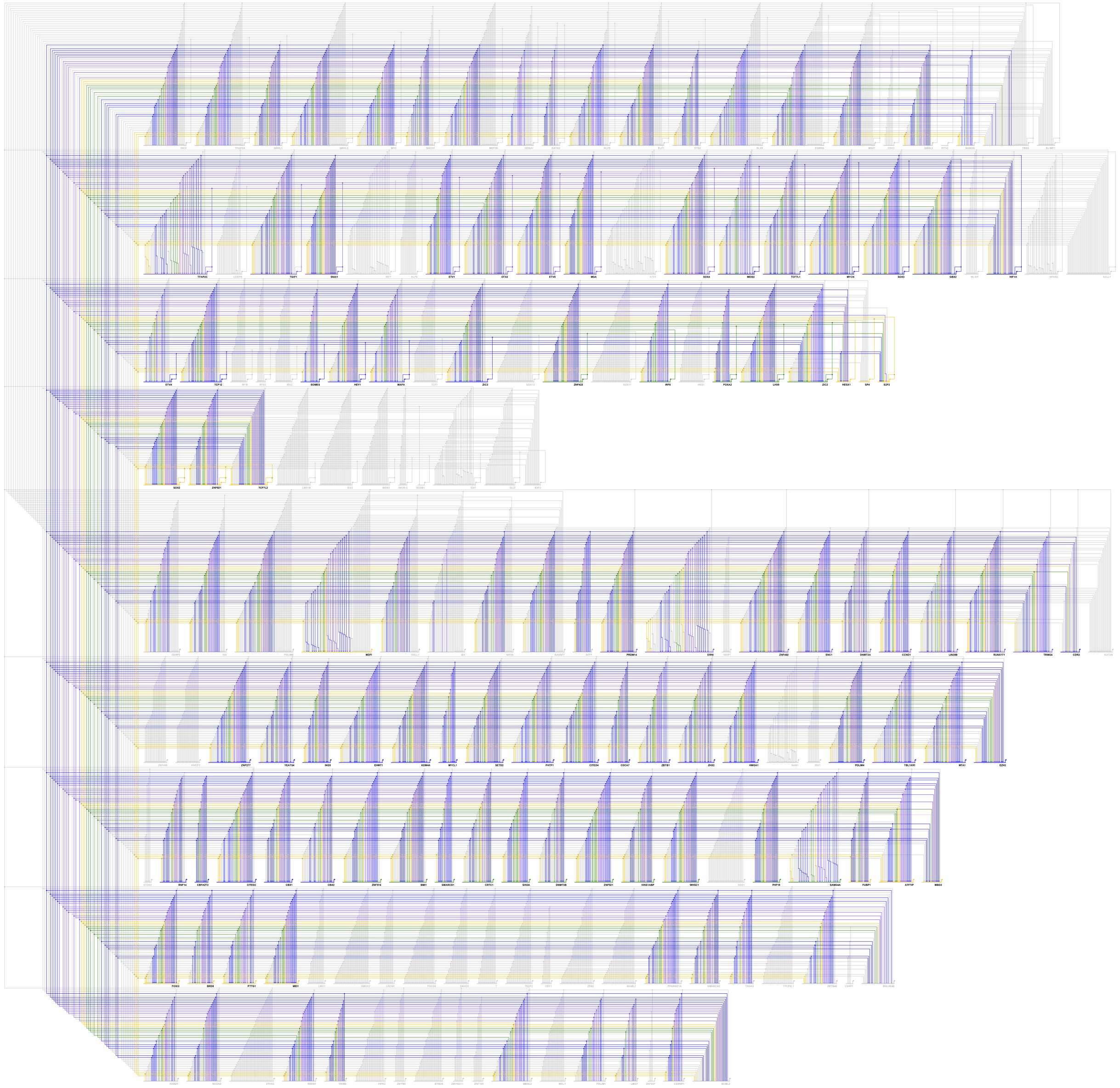

0, 1, 3, 5, 7, 9, 12h

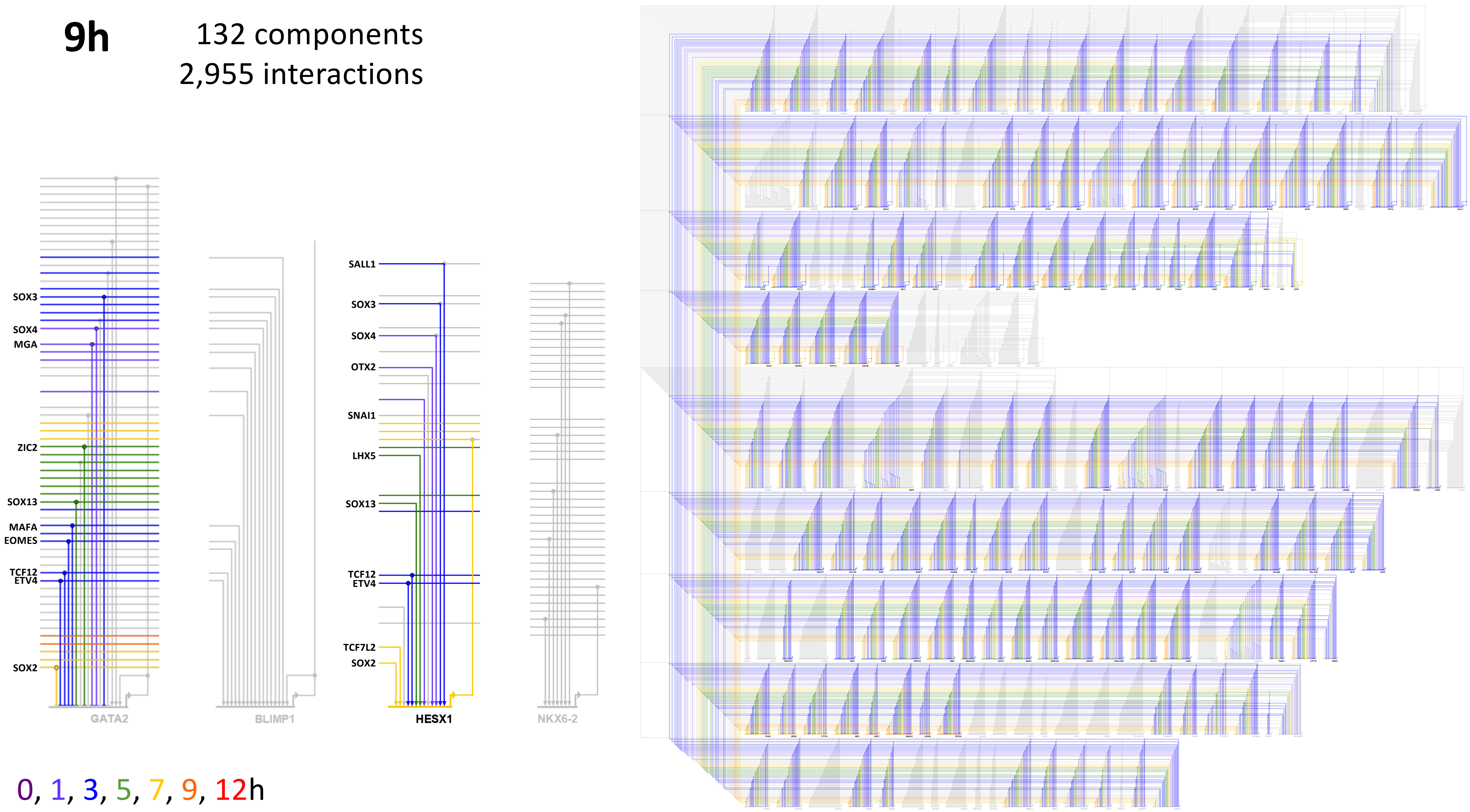
9h
132 components
2,955 interactions
SOX3
SOX4
MGA
ZIC2
SOX13
MAFA
EOMES
TCF12
ETV4
SOX2
GATA2
BLIMP1
SALL1
SOX3
SOX4
OTX2
SNAI1
LHX5
SOX13
TCF12
ETV4
TCF7L2
SOX2
HESX1
NKX6-2
0, 1, 3, 5, 7, 9, 12h

12h

143 components
3,322 interactions

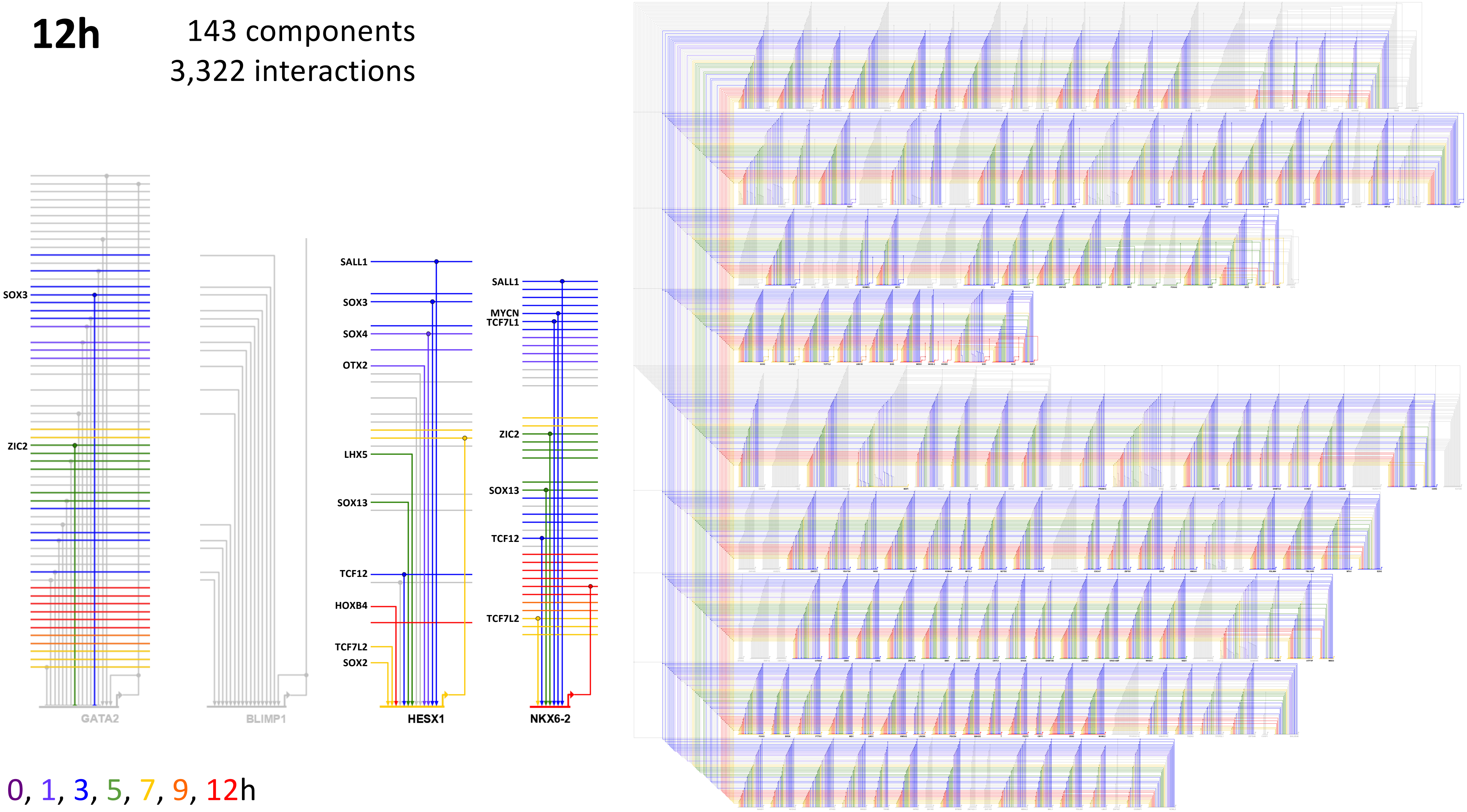

0, 1, 3, 5, 7, 9, 12h

All

175 components
5,614 interactions

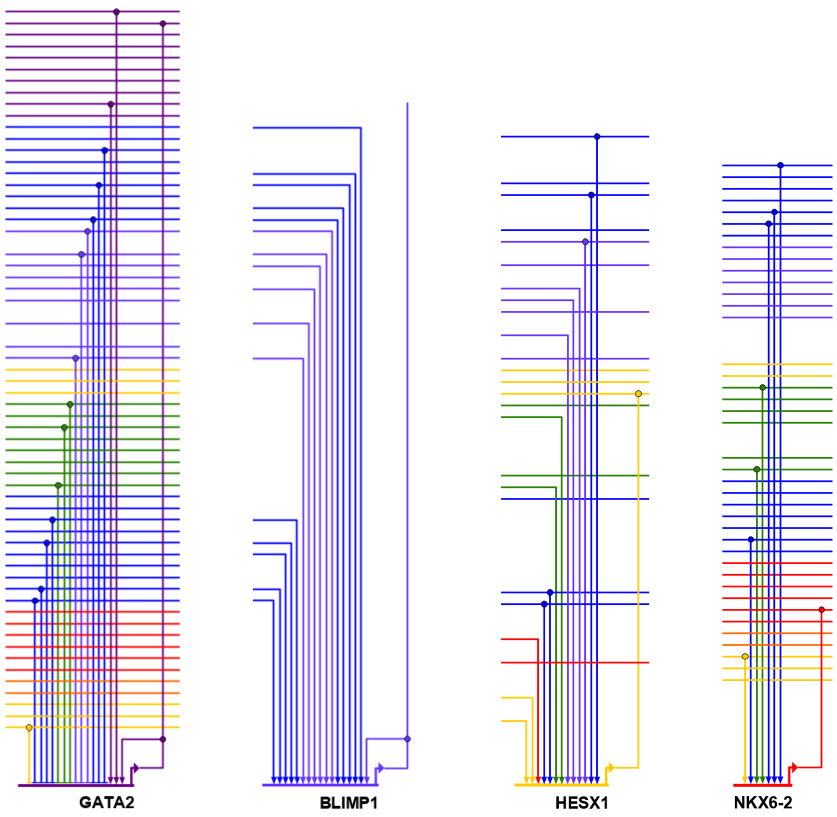

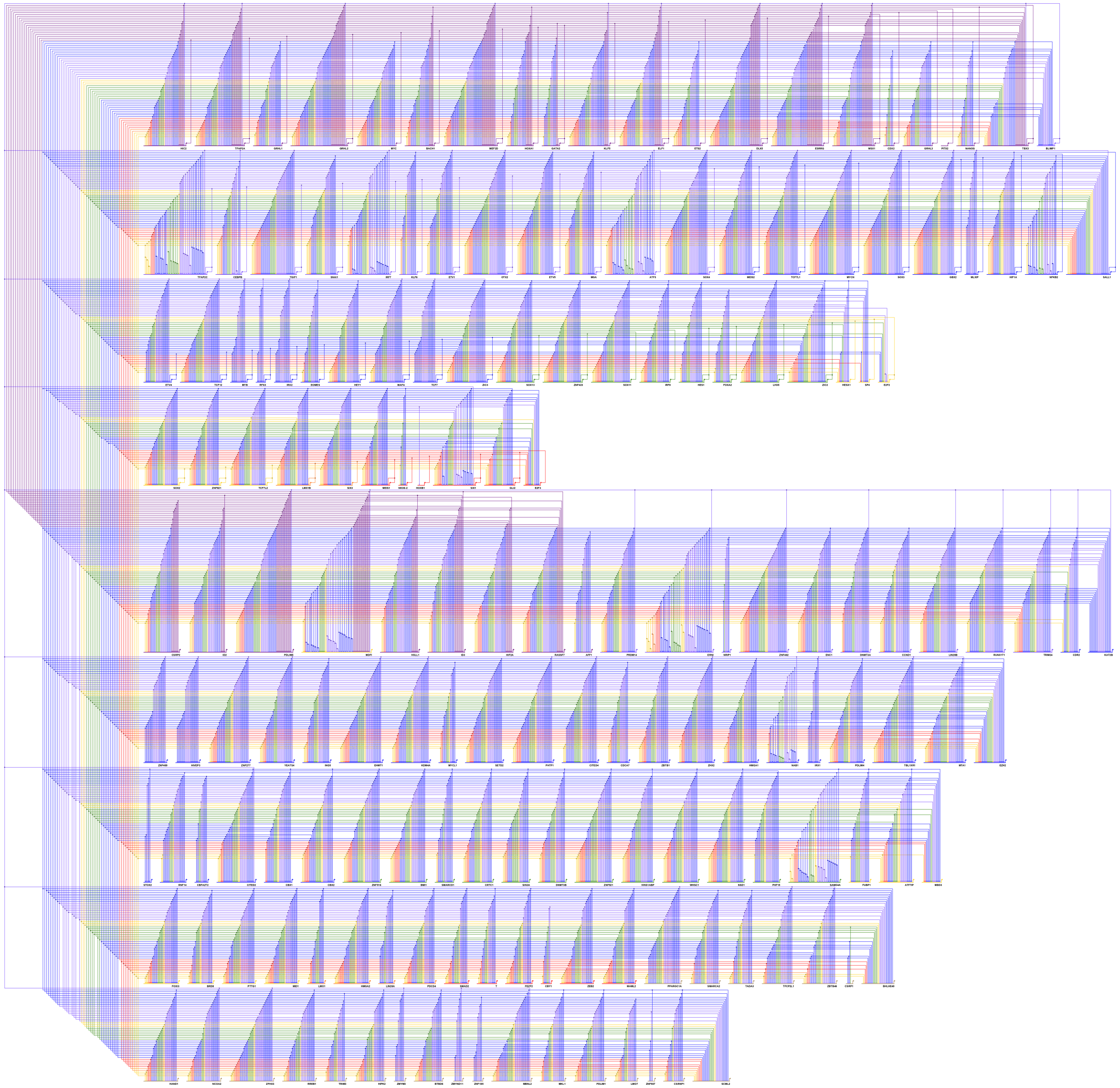

0, 1, 3, 5, 7, 9, 12h
